# A web-based decision aid for shared decision making in pelvic organ prolapse: the SHADE-POP trial

**DOI:** 10.1007/s00192-022-05405-0

**Published:** 2022-11-15

**Authors:** Larissa Esmeralda Drost, Marjan Stegeman, Maria B. E. Gerritse, Arie Franx, M. Caroline Vos, On behalf of the SHADE-POP study group, Romy E. D. Lamers, Nicole P. M. Ezendam, Anika Dam, Jan Schrickx, Heidy F. van Wijk

**Affiliations:** 1 Department of Obstetrics and Gynaecology, Elisabeth-Tweesteden Hospital, PO Box 90151, 5000 LC Tilburg, the Netherlands; 2 Department of Obstetrics and Gynaecology, Erasmus Medical Center, Rotterdam, the Netherlands; 3 Department of Obstetrics and Gynaecology, Gelderse Vallei Hospital, Ede, the Netherlands; 4 Department of Urology, UMC Utrecht, Utrecht, the Netherlands; 5 CoRPS—Center of Research on Psychology in Somatic diseases, Department of Medical and Clinical Psychology, Tilburg University, Tilburg, The Netherlands and Comprehensive Cancer Organisation, Utrecht, The Netherlands; 6 Department of Obstetrics and Gynaecology, VieCuri Medical Center, Venlo, the Netherlands; 7 Department of Obstetrics and Gynaecology, Rivas Beatrix Hospital, Gorinchem, The Netherlands; 8 Department of Obstetrics and Gynaecology, Bravis Hospital, Roosendaal, The Netherlands

**Keywords:** Shared decision making, Decision aid, Patient participation, Pelvic organ prolapse

## Abstract

**Introduction and hypothesis:**

Among women worldwide, pelvic organ prolapse (POP) is a common problem. There are three different treatment options for POP: pelvic floor muscle therapy, pessary treatment and prolapse surgery. As none of the three treatment options is clearly superior, shared decision making (SDM) is very important. A decision aid (DA) is known to facilitate patient participation and SDM. We hypothesise that the use of a web-based DA for POP increases patients’ satisfaction with information and care and reduces decisional conflict.

**Methods:**

This two-arm, multicentre, cluster randomised controlled trial was performed in women with POP in five different Dutch hospitals. The control group received usual care (UC) and the intervention group received the DA in addition to UC. Primary outcome measures were satisfaction with treatment decision making and satisfaction with information. Analyses were performed using independent sample *t* tests, Chi-squared tests, and multilevel linear regression analyses.

**Results:**

Between the DA group (*n*=40) and the UC group (*n*=56) no differences were found concerning patients’ satisfaction with information, with scores of 45.63 and 46.14 out of 50 respectively (*p*=0.67). Also, no differences were found concerning the perceived role in decision making, as patients scored 46.83 in the DA group and 46.41 in the UC group, out of a maximum of 54 (*n*=0.81).

**Conclusions:**

No differences were found concerning patients’ satisfaction with information and treatment decision making between the DA and UC. However, both groups scored high on the questionnaires, which suggests that the decision process is already of high quality.

## Introduction

Among women worldwide, pelvic organ prolapse (POP) is a common problem and can have major effects on quality of life. POP is defined as the descent of the anterior vaginal wall, posterior vaginal wall, the uterus (cervix), or the apex of the vagina (vaginal vault after hysterectomy) [1]. In the Netherlands, 11% of women aged between 45 and 84 years experience symptoms of POP [2]. POP symptoms include urinary, prolapse and defecatory symptoms, and can result in impaired quality of life [3, 4]. Treatment options for POP consist of pelvic floor muscle therapy (PFMT), pessary treatment and prolapse surgery, and none of these is clearly superior [5-8].

Decision making regarding treatment for POP is affected by many factors and can be challenging. Because no treatment option is superior, the current Dutch guideline recommends clinicians to discuss all treatment options with the patient [9]. Involving patients in this decision-making process is known as shared decision making (SDM). SDM is known to improve patient satisfaction and result in less decisional conflict [10]. However, the guideline on POP does not provide guidance on how to achieve SDM.

A decision aid (DA) is a way of facilitating SDM and can be helpful in clinical practice to support patient-centred care informed by the best evidence [11]. DAs improve patients’ knowledge of risks and benefits and make patients feel better informed and clearer about their values [12]. To our knowledge, only two small studies on the effect of online DAs for women with POP have been reported so far [13, 14].

To support SDM in women with symptomatic POP, an online DA for POP using a Delphi consensus procedure was developed. This DA provides patients with information on POP and contains value clarification exercises (VCEs) to provide insight in the values individuals attach to the consequences of the different treatment options. VCEs are known to decrease decisional conflict and values-incongruent choices [15]. The online aspect of the DA allows for availability at any place and time for both the patient and the clinician [16]. We hypothesised that the use of this DA for POP might reduce decisional conflict and increase patient satisfaction with information and care.

## Materials and methods

This two-arm multicentre cluster randomised controlled trial (RCT) was performed to evaluate the effects of an online DA on women with symptomatic POP. The study was approved by the Medical Ethical Research Committee Brabant, Tilburg, the Netherlands (NW 2015-62). The trial was registered as the SHAred DEcision making in Pelvic Organ Prolapse (SHADE-POP) trial, NL 55737.028.15. The CONSORT statement for reporting an RCT was followed [17].

### Study population and recruitment

Women with symptomatic POP who opted for (new) treatment were included. Inclusion criteria included eligibility for at least two treatment options and being able to use a computer with the internet. All the participants in the study signed a written informed consent form. Exclusion criteria were a history of gynaecological cancer, no access to the internet, insufficient knowledge of the Dutch language, more than one prolapse surgical procedure in the past, prolapse surgery in the past 2 years or participation in another study [18].

Pre-randomisation took place on a hospital level to determine whether patients of a certain hospital would receive usual care (UC) or UC with the DA. Randomisation was performed by a researcher not involved in the study and blinded to the identity of the hospitals. Because of the nature of the study patients and clinicians could not be blinded to the use of the DA. Cluster randomisation was chosen to prevent clinicians' knowledge of the DA and its implications from contaminating UC.

### Intervention and procedures

Women who were identified as eligible during their clinical visit sent the signed informed consent form to the Profiles Registry (www.profilesregistry.nl). After handing over the informed consent form to the patient, the clinician filled in the clinician version of the SDM-Q-9, the SDM-Q-Doc. The patients in the control arm received the usual information about POP and the possible treatment options, as they would normally receive during the first consultation (leaflets on paper and additional verbal information). The patients in the intervention group received UC including the leaflets on paper and additionally were provided with a code to access the DA. This DA informs patients by giving an overview of the treatment options, including the advantages and disadvantages of each one and clarifies patient preferences by VCEs [19]. Patients receive a results form showing the patients’ preferences for the specific treatment options, which can be taken to the next consultation. Questionnaires were sent by the Profiles Registry at four moments in time: a baseline questionnaire at 2 weeks after the decision was made but before initial treatment was started, and follow-up questionnaires at 6 months, 12 months and 24 months after baseline.

### Measures

To evaluate satisfaction with SDM, information and care the Shared Decision-Making Questionnaire (SDM-Q-9), the Satisfaction with Cancer Information Profile (SCIP-B) and the Patient Satisfaction Questionnaire (PSQ-18) were used [20-23]. The Decisional Conflict Scale (DCS) was used to evaluate decisional conflict concerning the treatment choice [24, 25]. Symptoms and health-related quality of life were assessed by the Pelvic Floor Disability Index (PFDI-20), the Pelvic Floor Impact Questionnaire (PFIQ-7) and the POP/Urinary Incontinence Sexual Functioning Questionnaire (PISQ) [26-28]. The health status was evaluated by the EuroQol-5D [29]. Clinicians were requested to fill out the Shared Decision-Making Questionnaire for health care providers (SDM-Q-doc) [23]. All questionnaires consist of Likert scales of four to six items and are validated questionnaires. Furthermore, baseline characteristics and socio-economic variables were included. Table 1 gives an overview of which questionnaires were sent at the specific moments.

**Table 1:** Outcome measures

Outcome measure		Questionnaire	T1	T2	T3	T4
**Patient characteristics**						
Baseline characteristics			X			
Socio-economic variables			X			
**Evaluation of decision making**						
Decision making	Decisional conflict	Decisional conflict scale	X			
	Satisfaction with treatment choice	SDM-Q-9	X	X	X	X
Information	Satisfaction with information	SCIP-B	X			
Care	Satisfaction with care	PSQ-18	X	X	X	X
DA-use (DA group only)			X			
**Long-term effects and patient-reported outcomes**						
Treatment	Treatment satisfaction			X	X	X
	Treatment choice		X	X	X	X
	Decisional regret	Decision regret scale		X	X	X
Well-being	Health-related quality of life	PFDI-20	X	X	X	X
		PFIQ	X	X	X	X
		PISQ	X	X	X	X
	Health status	EuroQol-5D	X	X	X	X

*SDM-Q-9* Shared Decision-Making Questionnaire, *SCIP-B* Satisfaction with Cancer Information Profile, *PSQ-18* Patient Satisfaction Questionnaire, *PFDI-20* Pelvic Floor Disability Index, *PFIQ-7* Pelvic Floor Impact Questionnaire, *PISQ-12* POP/Urinary Incontinence Sexual Functioning Questionnaire

### Outcome measures

The primary outcomes of the study are satisfaction with treatment decision (making) and satisfaction with information. The secondary endpoints are decisional regret, satisfaction with care, decisional conflict and quality of life. Other endpoints are the (baseline) clinical characteristics and socio-economic variables as well as the treatment choice.

### Statistical analysis

The sample size of the study was determined by power analysis on one of the primary endpoints: what is the effect of a DA on satisfaction with treatment choice? The power calculation was modified to the patient version of the SDM-Q-9. A difference of 8 was considered as clinically relevant and the standard deviation as calculated from the article was set at 26.02 [23]. Alpha was set at 0.05 and power at 0.80. The attrition rate was expected to be 25%, as was seen in other studies in comparable populations [30]. The intra-class coefficient (ICC) was set at 0.1, to ensure both adequate power and an acceptable sample size. Sample size calculation resulted in a total of 332 women (166 per group) to achieve a power of 80% and this was increased to 415 in total to account for loss to follow-up.

The analyses were performed on an intention-to-treat basis, on the assumption that information provision in the DA group was different from that of the UC group because of the introduction of the DA regardless of the actual DA usage by patients. Tests were two-sided and *p* value <0.05 was considered statistically significant. To compare (baseline) patient characteristics *t* tests for continuous variables and Chi-squared tests for categorical variables were used. Multilevel linear regression analyses were performed to evaluate differences between the DA group and the UC group, to take into account the clustering at hospital level [31]. Two levels were identified: patients and hospitals. The model included the random intercept “hospital-level” and the dependent variables SDM-Q9 and SCIP-B. Multilevel linear regression analyses showed no effect of clustering on hospital level; therefore, only the results of the naïve analyses are included in the results section.

Statistical analysis was performed using IBM SPSS Statistics for Windows, version 25.0 (IBM, Armonk, NY, USA).

## Results

**Fig. 1:**
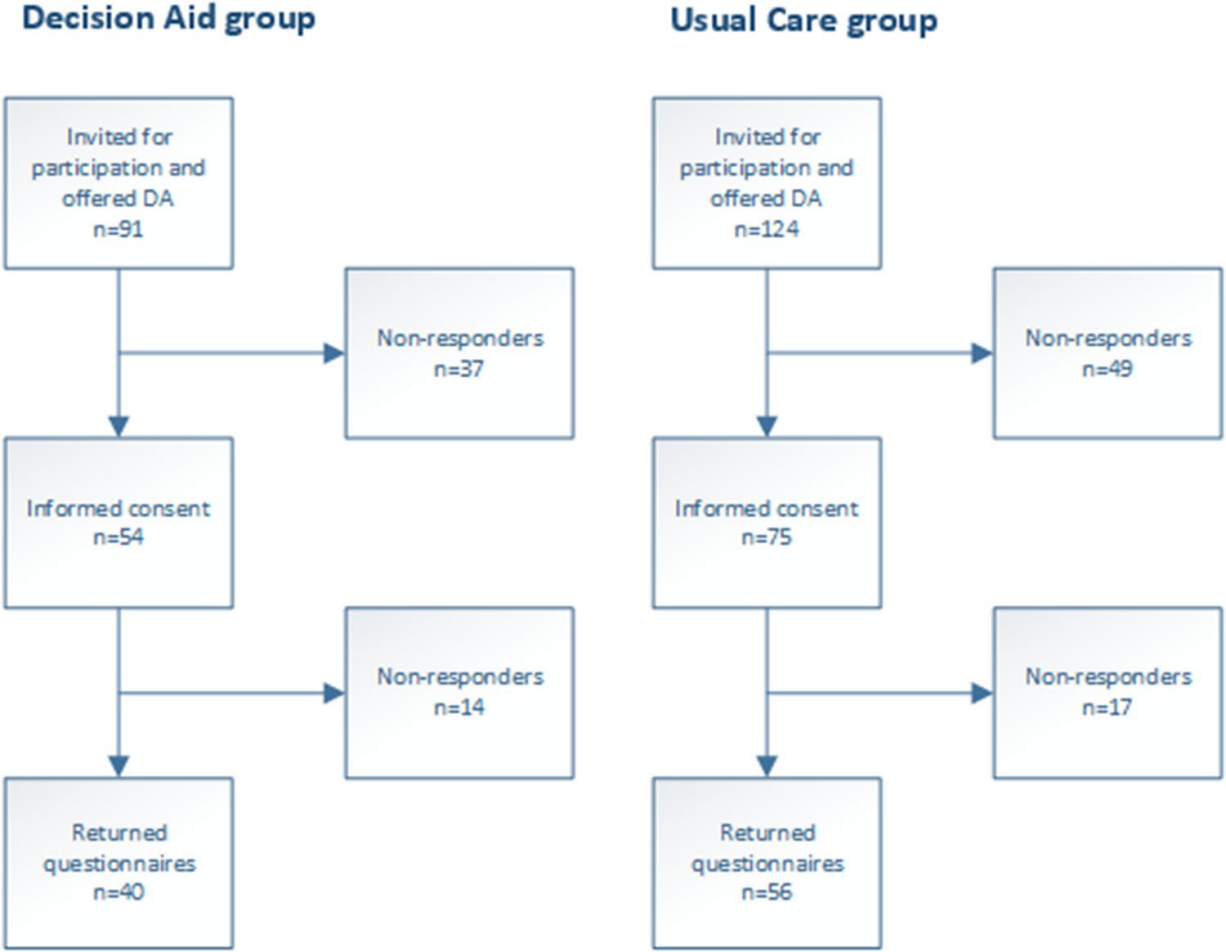
Flow-chart enrolment numbers

A total of 215 women with symptomatic POP were marked as eligible for inclusion in the study and received the patient information file. One hundred and twenty-nine patients signed a written consent form and were included in the study. Ninety-six patients (44.7%) completed (part of) the first set of questionnaires. In the intervention group, 100% of the patients were reported to have used the DA. A flowchart of the study, with enrolment numbers, is depicted in Fig. 1. Enrolment of patients was very difficult during the COVID-19 pandemic. After the pandemic, the DA was made available publicly; therefore, further continuation of the trial was not possible.

The mean age of responders at informed consent was 62.0 years. Age, educational level, BMI, parity, severity of complaints as defined by the PFDI-20, the PFIQ-7 and the Euroqol-5D were comparable between groups, as is shown in Tables 2 and 3. The PISQ-12 showed a greater impact on sexual functioning in the UC group (*p*=0.03). The number of patients enrolled per hospital varied between 7 and 39. One hospital switched from a UC group to a DA group after the inclusion of 39 patients.

**Table 2:** Patient characteristics

	Total (*n*=96)	DA group (*n*=40)	UC group (*n*=56)	*p* value
Age in years, mean (SD)	62.0 (7.8)	62.1 (7.7)	61.9 (8.0)	0.92
Educational level, *n* (%)				0.52
Low	17 (18)	6 (15)	11 (20)	
Medium	51 (53)	24 (60)	27 (48)	
High	28 (29)	10 (25)	18 (32)	
BMI, mean (SD)	25.6 (3.5)	25.1 (2.6)	26.0 (4.0)	0.21
Parity, mean (SD)	2.42 (1.22)	2.55 (1.15)	2.32 (1.27)	0.37
Preferred treatment option, *n* (%)				0.53
Expectant management/PFMT	12 (13)	7 (18)	5 (9)	
Pessary	47 (49)	19 (48)	28 (50)	
Surgery	36 (38)	14(35)	22 (39)	
Other	1 (1)	0	1 (2)	

*DA* decision aid, *UC* usual care

**Table 3:** Results of questionnaires concerning patient characteristics

Outcome	Range of scores	DA group (*n*=40), mean (SD)	UC group (*n*=56), mean (SD)	*p* value
PFDI-20 (pelvic floor disability)	0–80	16.55 (8.92)	20.29 (12.60)	0.11
PFIQ-7 (pelvic floor impact)	21–84	27.23 (5.84)	29.23 (8.36)	0.19
PISQ-12^a^ (impact on sexual functioning)	12–60	45.55 (6.13)	48.76 (4.84)	0.03
Euroqol-5D (impact on daily activities)	5–25	6.88 (1.92)	6.59 (2.14)	0.50

*PFDI-20* Pelvic Floor Disability Index, *PFIQ-7* Pelvic Floor Impact Questionnaire, *PISQ-12* POP/Urinary Incontinence Sexual Functioning Questionnaire, *DA* decision aid, *UC* usual care

^a^22 respondents in the DA group, 34 respondents in the UC group

Analysis of the results for the two groups was by intention to treat. All patients in the DA group confirmed in the questionnaires that they had used the DA. The possibility that some patients in the UC group also used the DA because during the trial it became available online with free access cannot be excluded.

### Satisfaction with information and treatment decision making

Between the two groups, no differences were found concerning patients’ satisfaction with information and the satisfaction with treatment decision making (Table 4). Patients in the DA group and the UC group scored 45.63 and 46.14 respectively on the SCIP-B (satisfaction with information) questionnaire, out of a maximum of 50 (*p*=0.67). On the SDM-Q-9 (perceived role in decision making) questionnaire patients scored 46.83 in the DA group and 46.41 in the UC group, out of a maximum of 54 (*p*=0.80). On the SDM-Q-Doc (perceived role in decision making of the physician) an average of 48.86 was scored in the DA group, which is comparable with 47.50 in the UC group (*p*=0.07). Multilevel analysis for the SDM-Q-9 and SCIP-B questionnaires did not show any effect of clustering at hospital level and therefore results are not shown. Within the hospital crossing over from the UC group to the DA group no differences were observed in the scores of the SCIP-B questionnaire (46.18 vs 45.24, *p*=0.54) and the SDM-Q-9 questionnaire (45.84 vs 44.95, *p*=0.70).

**Table 4:** Results of questionnaires

Outcome	Range of scores	DA group (*n*=40), mean (SD)	UC group (*n*=56), mean (SD)	*p* value
SCIP-B (satisfaction with information)	10–50	45.63 (5.72)	46.14 (6.07)	0.67
SDM-Q-9 (perceived role in decision making)	9–54	46.83 (6.98)	46.41 (8.69)	0.80
SDM-Q-Doc^a^ (perceived role in decision making of the physician)	9–54	48.86 (2.42)	47.50 (3.88)	0.07
PSQ-18 (patient satisfaction)	18–90	69.05 (7.33)	71.11 (7.69)	0.19
DCS-16 (decisional conflict)	16–80	29.33 (8.66)	29.63 (10.87)	0.89

*SCIP-B* Satisfaction with Cancer Information Profile, *SDM-Q-9* Shared Decision-Making Questionnaire, *PSQ-18* Patient Satisfaction Questionnaire, *DCS-16* decisional conflict scale, *DA* decision aid, *UC* usual care

^a^36 respondents in the DA group, 50 respondents in the UC group

### Treatment choice

Concerning treatment choice, 12 patients opted for expectant management or PFMT (7 in the DA group, 5 in the UC group), 47 patients chose pessary treatment (19 in the DA group, 28 in the UC group) and 36 patients chose surgical treatment (14 in the DA group, 22 in the UC group). One patient was awaiting results from additional examinations before making a treatment choice. The treatment choice was not significantly affected by the DA (*p*=0.53; Table 2).

## Discussion

Between the DA group and the UC group, no differences were found concerning patients’ satisfaction with information and the perceived role in decision making. Also, there were no differences in treatment choice between the DA and UC. This does not match with the hypothesis that the use of a web-based DA might increase patients’ satisfaction with information and care and reduce decisional conflict. Several explanations for these findings can be considered.

### High baseline patient satisfaction

In general, DAs improve patient knowledge and make patients feel more knowledgeable and better informed [11, 12]. However, it can be seen in the UC group that our population of patients with POP is already very satisfied with information provision, scoring 46 out of 50 on the SCIP-B questionnaire. This may be caused by the patients being well educated and counselled effectively by the clinicians in the participating hospitals. The power of this study was calculated to the patient version of the SDM-Q-9, with a difference of 8 considered clinically relevant. Owing to the high score of our patient population on this questionnaire as well, being above 46 on a scale of 54, it is impossible to achieve this level of improvement. Therefore, we observe a ceiling effect.

### Small difference between DA and UC

Patients receive information orally from their clinician during the consultation and in the information leaflets provided. The DA for POP does provide identical information because the development was based on the existing leaflets. The most important difference between the leaflets and the DA is the presence of VCEs. This small difference in combination with the high baseline satisfaction as measured by the SCIP-B may be an explanation for the lack of improvement in the DA group.

### Aspects of the patient population

As POP is a health problem that mostly occurs in an elderly population, it is important to realise that age might affect the decision-making process. Even though DAs are also known to improve older adults’ knowledge and enhance their participation in SDM, it must also be noted that a lot is still unknown on the effectiveness of DAs for elderly patients [32].

### Effect on treatment choice

To a certain extent, studies in other diseases show that DAs reduce the number of people choosing elective surgery, in favour of more conservative options [12]. However, in this Cochrane review, the effect on treatment choice greatly varies between the different medical treatments and diseases. No effect of the DA on treatment choice was seen in our study, although our numbers are small.

### Implications of the data

POP is a lifelong condition for which several treatment options exist. However, none of the treatment options is clearly superior and each option has its advantages and disadvantages. As different aspects of the treatment are weighed differently by every woman and clinician, it is important to support women in the decision process. This is currently done during consultation by the clinician and by provision of information booklets. As the DA does not provide additional information and yet does contain VCEs, it may be a substitute for the information booklets in the future.

### Limitations

One of the limitations of this study is that patients have to be able to use a computer with internet access to use the web-based DA and to complete the online questionnaires for the study. Thus, the group of patients for whom the online aspect of the DA is a disadvantage is already excluded. This might have resulted in a more positive result in the DA group. Second, clinicians who are less positive about SDM might have been less motivated to participate in the study and might have included fewer patients. As we hypothesise that SDM is important in improving the satisfaction with information and care, this is the group in particular where improvement can be made. Even though we included clinicians on a hospital level, bias was not completely avoidable. Furthermore, the targeted sample size for this study was not reached, because of the COVID-19 pandemic, and so the study did not have adequate power to show small but possibly significant differences between the two groups. During the pandemic, the elective care was downgraded and fewer patients with POP consulted a clinician. Thereafter, the DA became publicly available and a risk of bias arose. The already high scores on the questionnaires concerning patient satisfaction with information and treatment decision making may have resulted in a ceiling effect on the outcome measures.

## Conclusion

In conclusion, it can be stated that patients are already very satisfied with treatment decision making for POP and the provided information regarding the decision. In this study, a web-based DA for women with POP did not result in more satisfied patients concerning treatment decision making or information provision in a group of patients with adequate digital literacy. Further research should investigate which subgroup of patients may benefit and whether the use of the DA at other moments in the diagnostic process, such as during a visit to the general practitioner or prior to the consultation with the gynaecologist, might improve SDM. Moreover, more qualitative insights into the decision and treatment processes may be helpful.
